# Macroporous polymers prepared *via* frozen UV polymerization of the emulsion-templates stabilized by a low amount of surfactant

**DOI:** 10.1039/c8ra01000e

**Published:** 2018-03-13

**Authors:** Xiaoxing Fan, Shengmiao Zhang, Yun Zhu, Jianding Chen

**Affiliations:** Shanghai Key Laboratory of Advanced Polymeric Materials, School of Materials Science and Engineering, East China University of Science and Technology Shanghai 200237 China shmzhang@ecust.edu.cn

## Abstract

Macroporous polymers based on high internal phase emulsions (HIPEs) possess tunable porous structures and device shapes, and these characteristics make it possible for it to be applied in many fields. However, such materials also demonstrate undesirable properties, such as their brittleness and chalkiness, due to a great amount of surfactant required (5.0–50.0%, relative to the external phase) to realize the transformation from HIPEs to macroporous polymers (polyHIPEs). Herein, O/W HIPEs stabilized by a small amount (as low as 0.1 wt%, relative to the external phase) of commercial surfactant were prepared by magnetic stirring and subsequently homogenizing, and well-defined polyHIPEs were obtained through frozen UV polymerization of these HIPEs. In this process, the prepared HIPE was squeezed out by an injector and frozen at once, which effectively prevented the coalescence of internal phase. Then a 365 nm UV light was utilized to initiate the polymerization and the temperature was kept at −20 °C in order to avoid the melting of the frozen HIPE. After the polymerization, samples, having a typical polyHIPE structure, were obtained. Besides, the original monomer, surfactant and the oil (internal phase) were respectively replaced, and well-defined polyHIPEs could still be obtained. All the results suggested that frozen UV polymerization of HIPEs was an effective and universal approach to produce polyHIPEs with a low amount of surfactant.

## Abstract 2

PolyHIPE is prepared *via* frozen UV polymerization of the HIPE stabilized by a tiny amount (*e.g.* 0.1 wt%) of surfactant.

## Graphical abstract

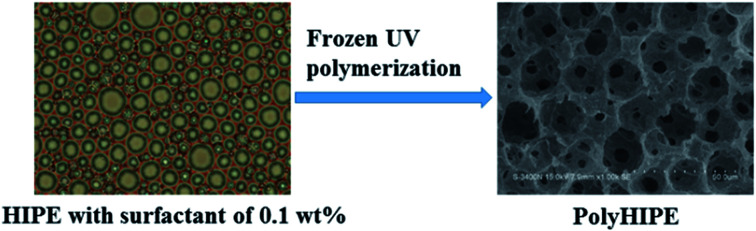

## Introduction

Due to their distinctive skeleton structure and remarkable properties, macroporous polymers are considered to have great potential applications in many fields, such as gas storage,^1^ ion adsorption,^2–4^ controlled release^5–9^ and support for catalyst.^10,11^ Emulsion templating is an attractive method for producing macroporous polymers, as it allows a high level of control over the porosity and pore size in the final materials.^12–15^ The emulsion templating method commonly involves the preparation of a high internal phase emulsion (HIPE) and the subsequent polymerization of its external phase (the continuous phase) to receive a porous material (polyHIPE).^16–19^ HIPE is a highly viscous, paste-like emulsion in which the internal phase (the dispersed phase) volume fraction occupies greater than 74%.^20–22^ Its dispersed phase (droplets) serves as the template of pores and the main question turns to be how to keep them stable without coalescence. Under the circumstance, relevant surfactants are usually necessary to stable the HIPE by spreading on the water and oil interface. So far, several typical surfactants, such as Tween85,^23^ Triton X-100,^24^ Triton X-405,^25^ Span80 (ref. 26 and 27) and block copolymer surfactant,^28^ are used frequently. However, corresponding to the existence of considerable dispersed phase, large amounts (5–50%)^29^ of surfactants are needed to meet the requirement of huge water and oil interface, which provides the HIPE with enough stability during the polymerization. However, the great employment of surfactant also causes some extra negative influences. The residual surfactant existing whatever in the surface or inner of the matrix, will make the composition of the final porous material more complex and increase its toxicity, which undoubtedly limits its application in the field requiring demanding environments, such as biomedicine.^30^ Besides, surfactant is universally regarded as the major influence factor of polyHIPEs' poor mechanical properties, such as their brittleness and chalkiness.^31^ Last but not least, it significantly increases the cost of raw material due to the mass application.

In order to solve the problems caused by large amounts of surfactant, many great efforts have been done. One is to replace surfactant with nanoparticles, such as silica particles^32,33^ titania particles,^23,34^ copolymer particles,^11,35–40^ ferroferric oxide^41^ and graphene oxide.^42^ In this process, the corresponding particles array on the water and oil interface closely and effectively prevent the touch of adjacent droplets.^43^ However, a vital limitation for this way was that it usually preferred to present a closed-cell porous structure,^44,45^ rather than open-cell structure stabilized by conventional surfactant.^46^ Although some specific particles could be utilized to prepare interconnected porous structure.^7,47^ On the other hand, a highly efficient surfactant was selected/synthesized to stabilize HIPEs. For example, cetyltrimethylammonium bromide (CTAB) of 0.3–2.0 wt% had been used to stabilize HIPEs,^48,49^ and well-defined interconnected polystyrene (PS) based polyHIPEs were obtained. Recently, Wang *et al.*^50^ synthesized a hyperbranched polyethylene having pendant sodium sulfonate groups (HBPE-SO_3_Na). With HBPE-SO_3_Na of 0.5–2.0 wt% as surfactant, stable HIPEs were obtained and utilized as templates to prepare high mechanical property porous PS. However, in these work, the successful reduction on the amount of surfactant strongly depended on the nature of surfactant. So far, a simple, feasible and universal method to reduce the usage of the surfactant in HIPEs is still highly desired.

In this work, well-defined porous poly(acrylamide) (PAM) based polyHIPEs were produced from a low amount (as low as 0.1 wt%) of Tween85 stabilized HIPEs. The prepared HIPEs were squeezed out in the shape of drops and frozen quickly through liquid nitrogen. And polyHIPEs were synthesized by frozen UV polymerization of these HIPEs. Moreover, by combining frozen UV polymerization and HIPE templating technique, a series of polyHIPEs were synthesized with different surfactants (*e.g.* Tween85, Tween60 or CTAB), dispersed phases (*e.g.* liquid paraffin or cyclohexane), and monomers (*e.g.* acrylamide (AM) or 4-vinylbenzenesulfonic sodium salt hydrate (SSNa)), which proved that frozen UV polymerization of HIPE was an effective and universal way to tremendously reduce the amount of surfactant used during the preparation of polyHIPEs.

## Experimental

### Materials

Acrylamide (AM), 4-vinylbenzenesulfonic acid sodium salt hydrate (SSNa), Tween85, Tween60 and cetyltrimethyl ammonium bromide (CTAB) were provided by Adamas Reagent Co., Ltd. *N*,*N*′-Methylene bisacrylamide (MBAM) was purchased from Sigma-Aldrich. 2,4,6-Trimethylbenzoyldiphenyl phosphine oxide (TPO), cyclohexane and ethanol were brought from Titan Scientific Co., Ltd (Shanghai, China). Liquid paraffin was obtained from Lingfeng Chemical Reagents Co. Ltd (Shanghai, China). All the reagents were used as received. Deionized water was used in this work.

### Synthesis of polyHIPEs

**Scheme 1:**
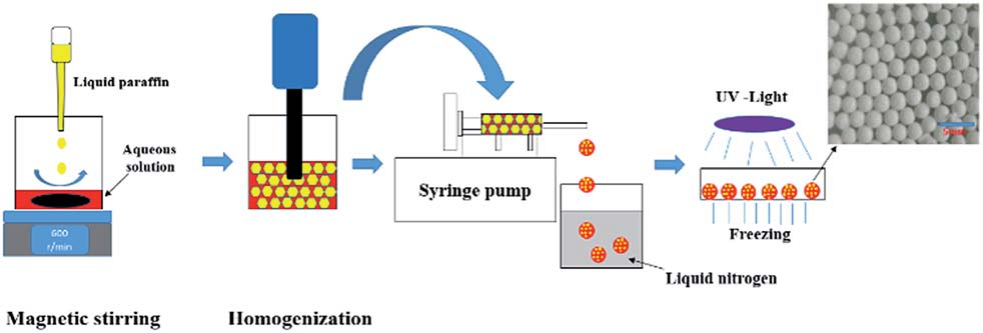
The preparation of HIPEs and polymer beads.

The preparation process was presented in Scheme 1. A certain amount of AM (0.8 g, 10 mmol) and MBAM (0.085 g, 0.5 mmol) were firstly dissolved in deionized water (2.4 ml), and then varied amount of Tween85 (0.1–1.0 wt%, relative to the water) was also added to the aqueous phase. Simultaneously, the photoinitiator TPO (3.0 wt%, relative to the monomers) was dispersed in 9.6 ml liquid paraffin under ultrasonic condition for 5 min. Subsequently, the prepared aqueous solution was poured into a 25 ml beaker and placed on the magnetic stirrer with a speed of 600 rpm. Then liquid paraffin was added dropwise to the aqueous solution at room temperature. After the addition of liquid paraffin was completed, the emulsion obtained was stirred for further 3 min.

After that, the prepared HIPE was transferred and treated with an Ultra Turrax T18 homogenizer with rotation speed of 4000, 8000, 12 000 and 16 000 rpm, respectively. The corresponding parameters of HIPEs and polyHIPEs were listed in Table 1. The HIPE was transferred into a 5 ml glass injector and squeezed out with a syringe pump and fell into liquid nitrogen, and were frozen immediately. Specially, what needs to be emphasized was that the operation mentioned above was conducted in dark because of the high activity of TPO. Next, the frozen beaded HIPEs were transferred to a Petri dish, and irradiated by 365 nm UV-light for 6 h in a −20 °C thermostatic ethanol bath. The resulting polyHIPE beads were immersed in cyclohexane for 4 days to remove the liquid paraffin, and then freeze-dried to constant. The prepared polyHIPEs were named as PAM-*x-y*, in which the *x* corresponding to the rotation speed, and the *y* represented the concentration of Tween85. For example, the sample PAM-12000-1 meant that the homogenizing rotation speed was 12 000 rpm and the concentration of Tween85 was 1.0 wt%.

**Table 1:** Parameters for HIPEs and polyHIPEs^a^

Sample	*S*_r_ (rpm)	*C*_t_ (wt%)	*V*_a_ (Pa s)	*D*_1_	*D*_2_
PAM-4000-1	4000	1.0	1.7	48.4 ± 10.2	30.1 ± 9.7
PAM-8000-1	8000	1.0	2.9	23.7 ± 6.4	15.3 ± 5.3
PAM-12000-1	12 000	1.0	4.0	10.5 ± 3.0	9.9 ± 3.3
PAM-16000-1	16 000	1.0	6.8	7.1 ± 3.9	6.9 ± 2.5
PAM-12000-0.1	12 000	0.1	1.5	27.2 ± 9.3	15.9 ± 9.8
PAM-12000-0.3	12 000	0.3	2.8	18.5 ± 6.0	11.9 ± 3.2
PAM-12000-0.6	12 000	0.6	3.5	11.4 ± 4.9	10.7 ± 4.1

a The internal phase volume fraction was 80%; the monomer concentration in continuous phase was 25 wt%; *S*_r_: the homogenizing rotation speed; *C*_t_: the Tween85 concentration (relative to the water); *V*_a_: the viscosity of HIPEs, specially the shear rate was 0.01 s^−1^ and the test temperature was 25 °C; *D*_1_: the average pore size calculated from SEM images; *D*_2_: the average size of droplets calculated from optical microscope photographs.

Additionally, SSNa (0.6 g, 4.0 mmol), cyclohexane (9.6 ml) and Tween60 (0.3 wt%, relative to water) or CTAB (0.3 wt%, relative to water), were respectively chosen as a replacement of monomer, dispersed phase, and surfactant to prepare corresponding HIPEs, while other conditions (Table 1) were unchanged.

### Characterizations

The microstructure of the HIPEs were observed by an inverted microscope (TE 2000-U, Nikon). To distinguish the continuous phase and dispersed phase, rhodamine B (0.5 wt%, relative to water) was added to the continuous phase, which has a good solubility in water while couldn't dissolve in liquid paraffin.

An analyzer of concentrated liquid dispersions (Turbiscan LAB Expert) was used to assess the stability of HIPEs by monitoring the change of backscattering in 30 min at room temperature. The wavelength of monochromatic light (*λ*) was 880 nm. The prepared HIPE was moved to a flat bottomed cylindrical glass tube (70 mm in height and 27.5 mm in diameter) before measurement.

Rotational rheometer (Thermo HAKKE, MARS3) was used to verify the effect of homogenizing rotation speed on the viscosity of HIPEs. The test temperature was 25 °C and the range of shear rate was between 0.01 and 50 s^−1^.

The morphology of the polyHIPE beads was observed with a Hitachi S-3400N SEM. A sample was cut with a surgical knife, and subsequently attached to conductive tap. Then the sample was coated with a layer of gold under vacuum condition. The average void size was calculated for at least 100 voids from SEM images through Image J software.

The FTIR spectrum was recorded by a Nicolet 5700 Fourier transform infrared spectrometer. Before scanning, the sample were dried to constant, and then treated with KBr together to get transparent tablets.

## Results and discussion

### Preparation of HIPEs with a small amount of surfactant

**Fig. 1:**
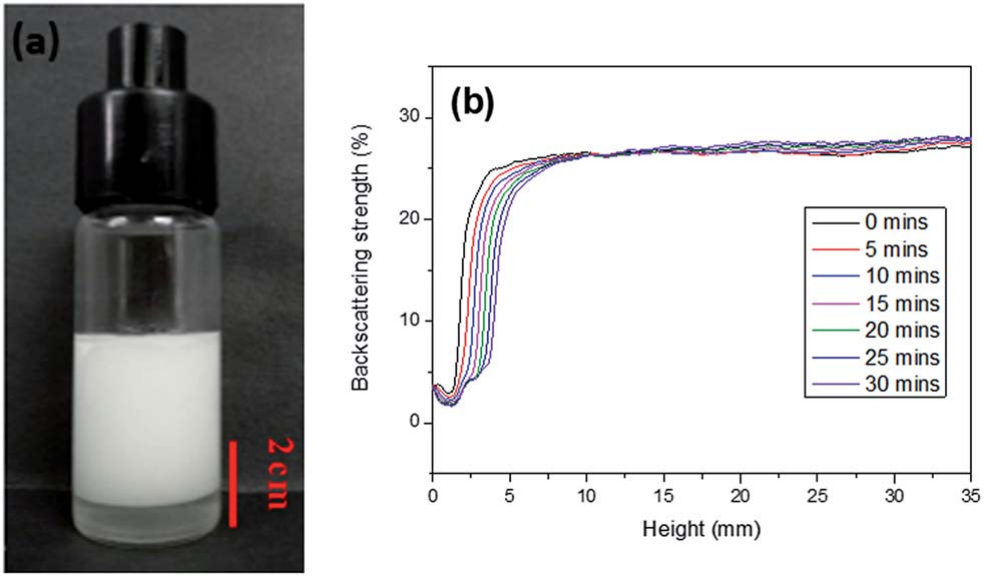
The O/W HIPE stabilized with 1.0 wt% Tween85 and emulsified by magnetic stirring. (a) Digital photograph of HIPE placed for 30 mins after prepared; (b) backscattering date of HIPE for different positions within 30 mins at 25 °C.

In this work, AM, MBAM and Tween85 dissolved in 2.4 ml water as continuous phase were stirred with a magnetic stirrer at 600 rpm in a 25 ml glass beaker. Then 9.6 ml liquid paraffin containing TPO (3.0 wt%, relative to monomers) as dispersed phase was added dropwise to the continuous phase. After all the paraffin was dropped over, stirring the HIPE for further 3 min. Finally, a milky-white HIPE was obtained. However, because only 1.0 wt% Tween85 was used (much lower than the surfactant amount used in previous HIPEs),^51^ the bottom of emulsion turned transparent from milky-white in several minutes (Fig. 1a). In attempt to further view the sedimentation procedure of the emulsion, the backscattering data of the prepared HIPE was detected by Turbiscan (Fig. 1b). It was found that the backscattering of HIPE decreased sharply in a short time within a certain height (below 10 mm). While for the height higher than 10 mm, the backscattering increased slightly with time. This represented a typical emulsion separation curve,^52^ and was consistent with the phenomenon observed by the naked eye (Fig. 1a), all of these meant that the HIPE stabilized with 1.0 wt% Tween85 was unstable, when it was emulsified solely by magnetic stirring.

**Fig. 2:**
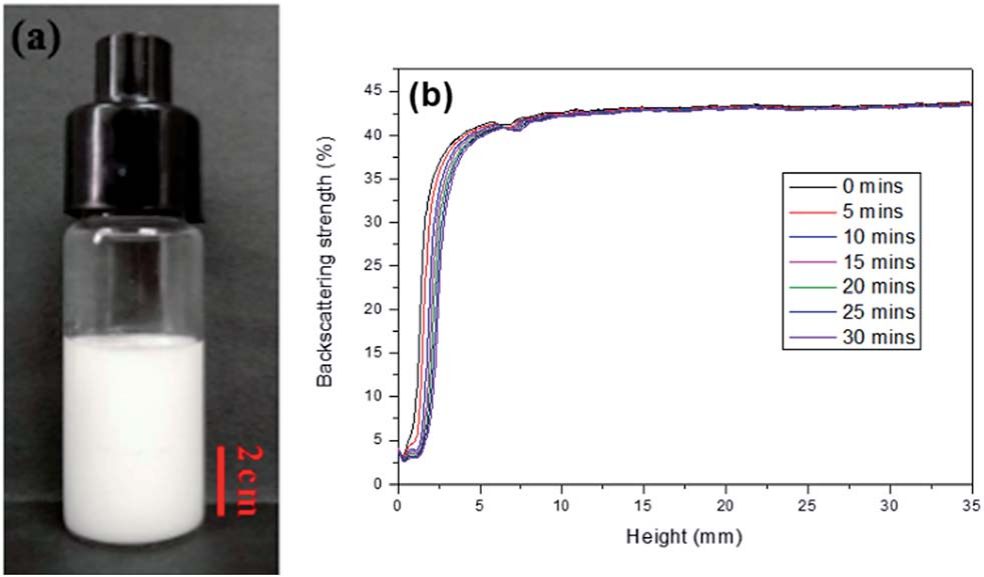
The O/W HIPE stabilized with Tween85 (1.0 wt%, relative to the water) and emulsified by a homogenizer (the rotation speed was 4000 rpm). (a) Digital photograph of HIPE placed for 30 mins after prepared; (b) backscattering date of HIPE for different position within 30 min at 25 °C.

**Fig. 3:**
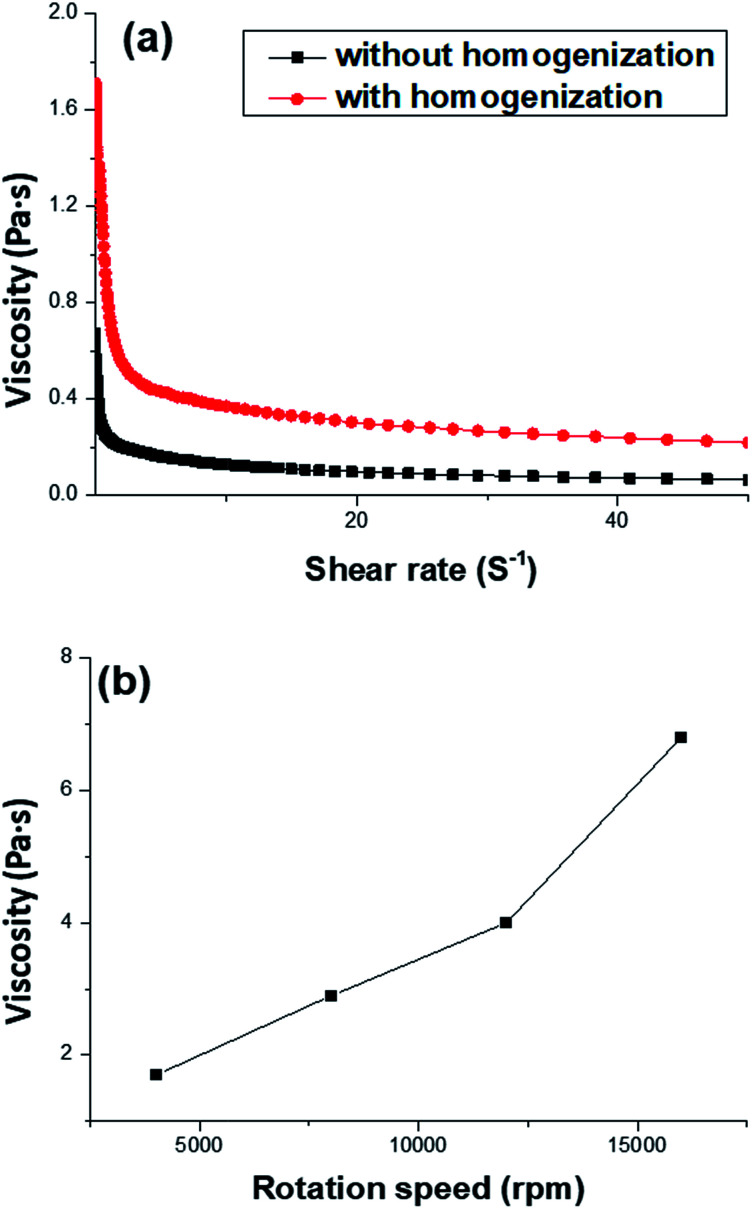
(a) Viscosity of HIPEs before and after homogenization respectively, the Tween85 content was 1.0 wt% relative to the water, the rotation speed during homogenizing was 4000 rpm. (b) Viscosity of HIPEs dealt with a homogenizer at different rotation speed, the Tween85 content was 1.0 wt% relative to the water. The shear rate of the rotational rheometer was 0.01 s^−1^ and the temperature was kept at 25 °C.

**Fig. 4:**
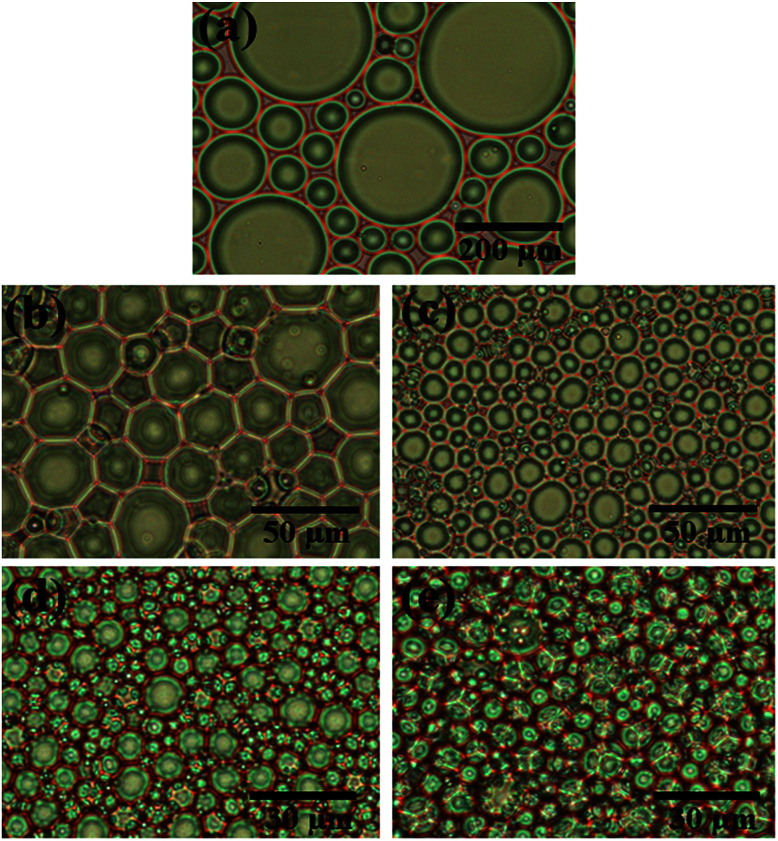
Inverted microscope photographs of HIPEs stabilized by Tween85 of 1.0 wt%. (a) Emulsified with a magnetic stirring of 600 rpm; (b–e) emulsified by a magnetic stirring of 600 rpm and followed by a homogenization at different rotation speed. The rotation speed during homogenization for (b) 4000 rpm, (c) 8000 rpm, (d) 12 000 rpm, and (e) 16 000 rpm.

To enhance the stability of the HIPE, the prepared emulsion was further emulsified with a homogenizer at rotation speed of 4000 rpm for 2 min. As shown in Fig. 2a, no obvious sedimentation was observed by the naked eye during 30 min after homogenization. In order to know more detail of the changes of the HIPE, the backscattering data of this emulsion was measured. It was confirmed that the stability of the emulsion was significantly enhanced after homogenization, although the backscattering slightly decreased at the bottom within 10 mm height (Fig. 2b). In addition, the backscattering data of the HIPE in the middle part was around 43%, which was much greater than that of the HIPE without homogenization (∼27%, Fig. 1b). This meant the HIPE with homogenization was much more stable than the emulsion without homogenization.^53^ This conclusion was also supported by the analysis of the HIPEs through rotational rheometer. As shown in Fig. 3a, the viscosity (0.6 Pa s at the shear rate of 0.01 s^−1^) of HIPE without homogenization was lower than that (1.7 Pa s at the shear rate of 0.01 s^−1^) of the HIPE with homogenization. This phenomenon could also be explained through the analysis of inverted microscope photographs of HIPEs. As shown in Fig. 4a, the dispersed phase (droplets) presented spherical shape with a broad size distribution. The large space between the adjacent droplets allowed the dispersed droplets to move easily. In addition, because of the small amount of Tween85 (1.0 wt%, relative to water), the adjacent droplets were inclined to reduce the oil–water interface area in the way of merging together and became larger droplets. This could also be used to explain why the HIPE emulsified by magnetic stirring was unstable and had low viscosity. While the HIPE, after homogenizing, had a much smaller size compared with that of the HIPE without homogenization (Fig. 4b–e). And the dispersed droplets tightly piled up together, which made it more difficult to move and exhibited higher viscosity. So the homogenization really improved the stability of the HIPE, and made it a potential emulsion-template candidate for the synthesis of polyHIPEs with UV polymerization.

### PolyHIPEs prepared *via* frozen UV polymerization of HIPEs

The HIPE was transferred to a 5 ml glass injector immediately after homogenization, and subsequently extruded to liquid nitrogen as beaded HIPEs with a syringe pump, as illustrated in Scheme 1. PolyHIPEs were successfully synthesized with frozen UV polymerization of the beaded HIPEs at −20 °C.

**Fig. 5:**
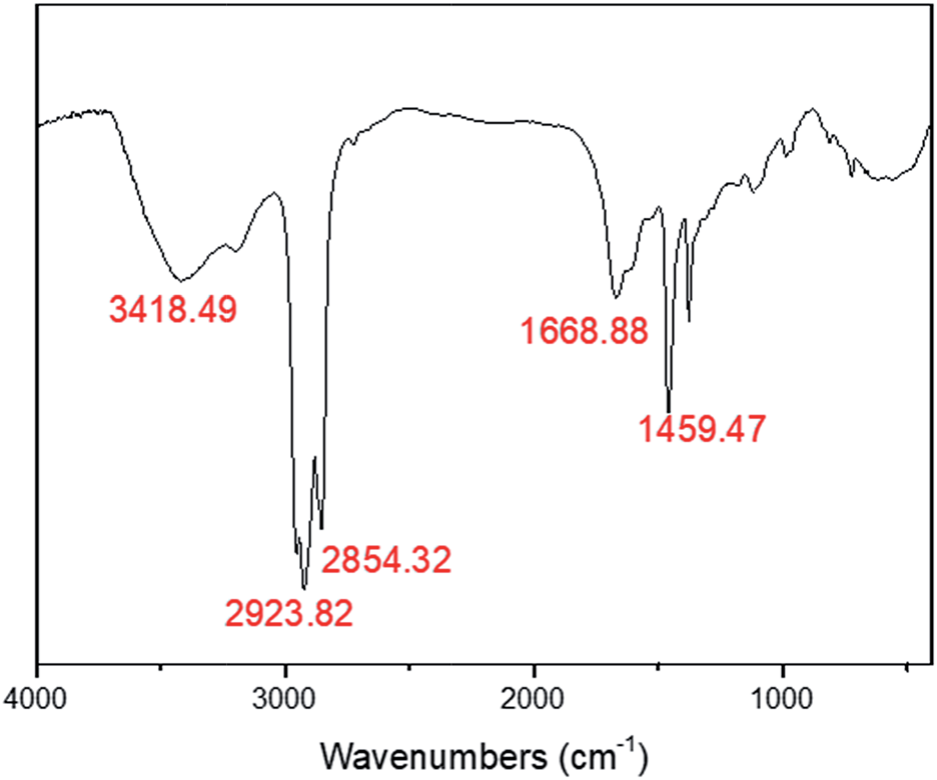
FT-IR spectra of PAM-4000-1.

As shown in Fig. 5, the FTIR spectra of the polyHIPE presented typical PAM structure characteristics. The peak of 3418.5 cm^−1^ was corresponded to the stretching vibrations of –NH; the band of 1668.9 cm^−1^ matched with the stretching vibrational absorption of –C=O. The typical absorption peak of –C–N could also be observed at 1459.4 cm^−1^. The strength of peak appeared in the wavenumbers of 2923.8 cm^−1^ and 2854.3 cm^−1^ was remarkable stronger than others, which was arose from the group of –CH_2_–.

**Fig. 6:**
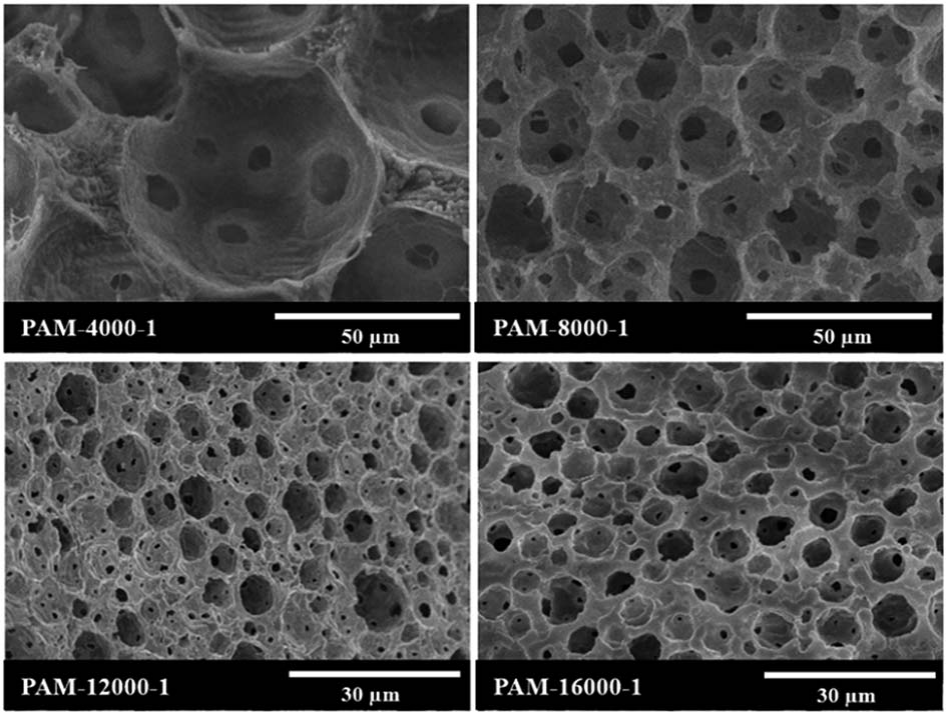
SEM images of polyHIPEs prepared with different rotation speeds, and the amount of Tween85 was 1.0 wt%.

In attempt to tune the morphology of the polyHIPEs, the HIPEs were prepared with Tween85 of 1.0 wt% and varied homogenizing rotation speeds in the range from 4000 to 16 000 rpm (Table 1), and the corresponding polyHIPEs were obtained. As shown in Fig. 6, the polyHIPE herein had a typical interconnected pore structure as those materials obtained by the conventional radical polymerization of large amount surfactants stabilized HIPEs.^54^

**Fig. 7:**
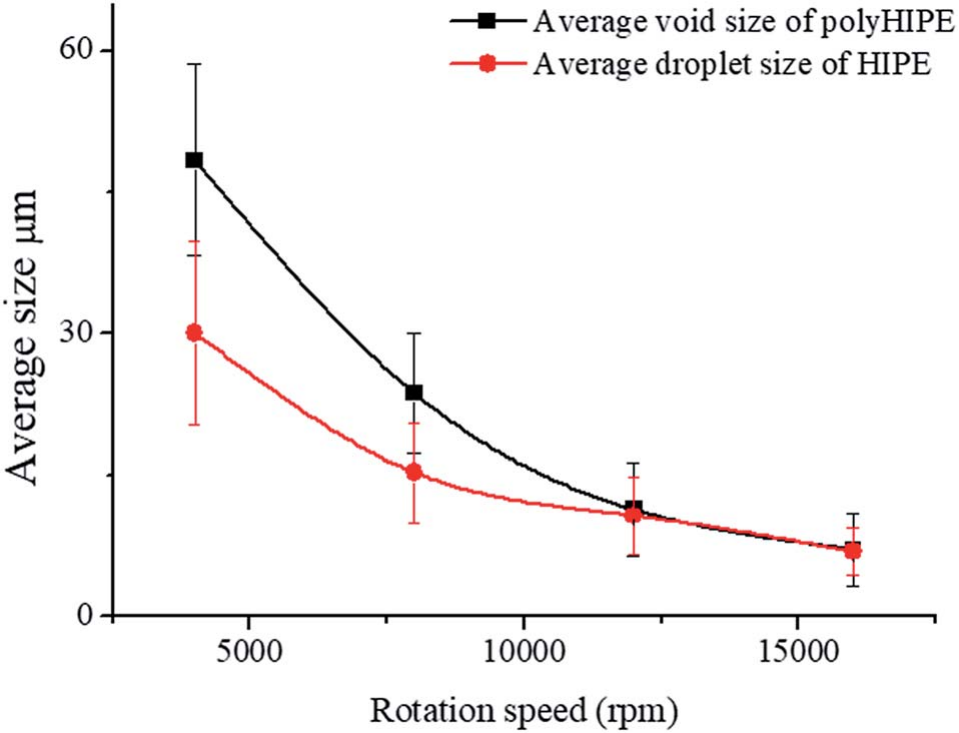
Average size calculated from inverted microscope photographs and SEM images at different rotation speed, the Tween85 concentration was 1.0 wt%.

The average void size of the resulting polyHIPEs decreased obviously with an increase of the rotation speed from 4000 to 12 000 rpm, and then decreased gradually with further increasing the rotation speed from 12 000 to 16 000 rpm (Fig. 7). This is due to the change of dispersed droplet size caused by varying the homogenizing rotation speed, since the voids are considered as the removal of the dispersed droplets of the polymerized HIPEs.^55^ As shown in Fig. 4b–e, 7, and Table 1, with an increase of the rotation speed, the average dispersed droplet size of HIPEs decreased significantly, and then decreased slightly. Moreover, with the increasing of the rotation speed, the droplets tightly piled together and the droplet shape turned polyhedron from spherical gradually, so it need more force for the droplets to move in the emulsion, and consequently increased the viscosity of the emulsion, and enhanced the emulsion stability.

**Fig. 8:**
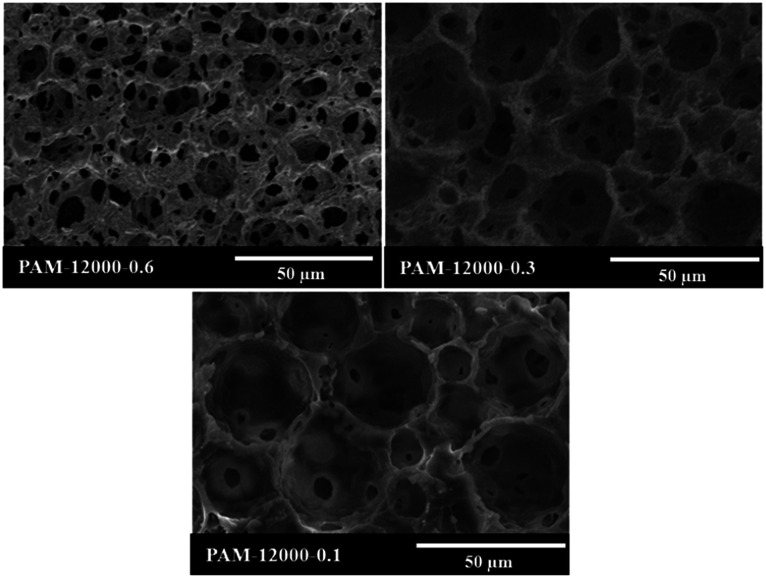
SEM images of polyHIPEs prepared with different amount of Tween85, the rotation speed was 12 000 rpm.

The aim of this work was to reduce the usage of surfactant during the preparation of polyHIPEs. To study the limits of the surfactant amount, HIPEs were prepared with homogenizing rotation speed at 12 000 rpm and Tween85 of 0.6, 0.3 and 0.1 wt%, respectively (Table 1). The corresponding polyHIPEs (PAM-12000-0.6, PAM-12000-0.3, and PAM-12000-0.1) were successfully produced by frozen UV polymerization of these HIPEs. As shown in Fig. 8, all these three polymers have a well-defined void structure as well as an obvious interconnected pore structure, even though the amount of Tween85 used to stabilize HIPE was as low as 0.1 wt%. In addition, we found that the amount surfactant had a great effect on the void and interconnected pore. The average void size corresponding to PAM-12000-0.1, PAM-12000-0.3, and PAM-12000-0.6 were 27.2, 18.5 and 11.4 μm respectively. The void size increased as the amount of surfactant decreased, this phenomenon could be attributed to coalescence between droplets. Less surfactant meant that the emulsion was less-stable, and larger droplets were formed at the expense of smaller ones. On the contrary, the reduction of surfactant was helpful for enhancing the polyHIPEs' mechanical properties, which led to less interconnected pores.

**Fig. 9:**
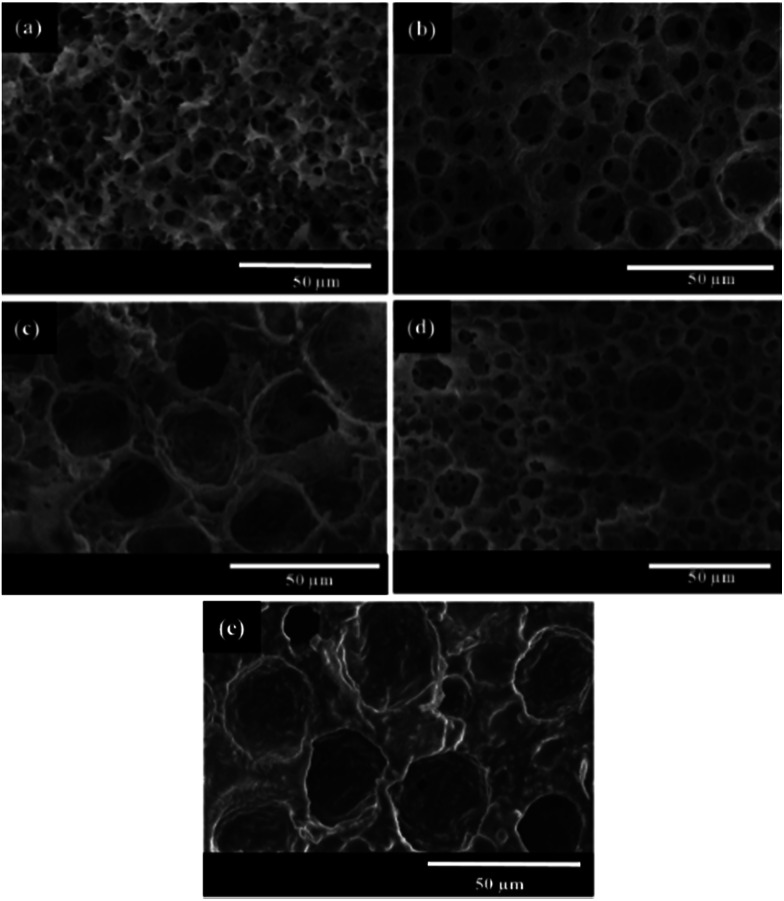
SEM images of the polyHIPEs prepared with different surfactants, dispersed phase and monomers. The homogenizing rotation speed was 12 000 rpm. (a) The HIPE stabilized with CTAB of 0.3 wt%, the dispersed phase was liquid paraffin, and the monomers were AM and MBAM; (b) the HIPE stabilized with Tween60 of 0.3 wt%, the dispersed phase was liquid paraffin, and the monomers were AM and MBAM; (c) the HIPE stabilized with Tween85 of 0.3 wt%, the dispersed phase was cyclohexane, and the monomers were AM and MBAM; (d) the HIPE stabilized with Tween85 of 0.3 wt%, the dispersed phase was liquid paraffin, and the monomers were SSNa and MBAM; (e) the HIPE stabilized with Tween85 of 0.1 wt%, the dispersed phase was liquid paraffin, and the monomers were SSNa and MBAM.

In attempt to prove the universality of frozen UV polymerization in reducing the usage of surfactant, HIPEs were prepared with CTAB and Tween60 of 0.3 wt%, respectively. And the PAM-based polyHIPEs were also synthesized by frozen UV polymerization of these HIPEs (Fig. 9a and b). Replacing liquid paraffin with cyclohexane, a PAM-based polyHIPE was obtained with the cyclohexane-in-water HIPE that was stabilized by Tween85 of 0.3 wt% (Fig. 9c). Alternatively, a PSS-based polyHIPE was synthesized by frozen UV polymerization of the HIPE stabilized by Tween85 of 0.3 and 0.1 wt% respectively (Fig. 9d and e). These results meant that frozen UV polymerization of HIPEs was an effective and universal method for synthesis of polyHIPEs with a low amount of surfactant.

## Conclusions

A series of porous PAM were successfully synthesized by frozen UV polymerization of the paraffin-in-water HIPEs stabilized by a low amount of Tween85 surfactant (as low as 0.1 wt%). These PAM-based polyHIPEs had a well-defined open-cell and tuneable structure. Compared with the great amount of surfactant (5–50%) required in conventional procedure of preparing polyHIPEs, frozen UV polymerization of HIPEs significantly reduced the amount of surfactant needed for synthesis of polyHIPEs. Besides, respectively replacing AM, Tween85, and paraffin with SSNa, Tween60 (or CTAB), and cyclohexane, typical porous polyHIPEs could still be obtained. All the results suggest that frozen UV polymerization of HIPEs is an effectively and universal approach to produce polyHIPEs with a low amount of common surfactant.

## Conflicts of interest

There are no conflicts to declare.

## Supplementary Material

## References

[cit1] Makal T. A., Li J. R., Lu W., Zhou H. C. (2012). Chem. Soc. Rev..

[cit2] Zhu Y., Zheng Y., Wang F., Wang A. (2016). Chem. Eng. J..

[cit3] Pan J., Zeng J., Cao Q., Gao H., Gen Y., Peng Y., Dai X., Yan Y. (2016). Chem. Eng. J..

[cit4] Moghbeli M. R., Khajeh A., Alikhani M. (2017). Chem. Eng. J..

[cit5] Hu Y., Gao H., Du Z., Liu Y., Yang Y., Wang C. (2015). J. Mater. Chem. B.

[cit6] McGann C. L., Streifel B. C., Lundin J. G., Wynne J. H. (2017). Polymer.

[cit7] Hu Y., Gu X., Yang Y., Huang J., Hu M., Chen W., Tong Z., Wang C. (2014). ACS Appl. Mater. Interfaces.

[cit8] Moglia R., Whitely M., Brooks M., Robinson J., Pishko M., Cosgriff-Herhandez E. (2014). Macromol. Rapid Commun..

[cit9] Grant N. C., Cooper A. I., Zhang H. (2010). ACS Appl. Mater. Interfaces.

[cit10] Zhu Y., Hua Y., Zhang S., Wang Y., Chen J. (2015). J. Polym. Res..

[cit11] Yi F., Xu F., Gao Y., Li H., Chen D. (2015). RSC Adv..

[cit12] Zhang H., Cooper A. I. (2005). Soft Matter.

[cit13] Langford C. R., Johnson D. W., Cameron N. R. (2014). Polym. Chem..

[cit14] Pulko I., Krajnc P. (2012). Macromol. Rapid Commun..

[cit15] Carnachan R. J., Bokhari M., Przyborski S. A., Cameron N. R. (2006). Soft Matter.

[cit16] Caldwell S., Johnson D. W., Didsbury M. P., Murray B. A., Wu J. J., Przyborski S. A., Cameron N. R. (2012). Soft Matter.

[cit17] Luo Y., Wang A. N., Gao X. (2012). Soft Matter.

[cit18] Kovačič S., Silverstein M. S. (2016). Macromol. Rapid Commun..

[cit19] Butle R., Davies C. M., Cooper A. I. (2001). Adv. Mater..

[cit20] Gurevitch I., Silverstein M. S. (2011). Macromolecules.

[cit21] Deleuze H., Faivre R., Herroguez V. (2002). Chem. Commun..

[cit22] Zhang H., Zhu Y., Chen J., Zhang S. (2017). J. Polym. Sci., Part A: Polym. Chem..

[cit23] Hua Y., Zhang S., Zhu Y., Chu Y., Chen J. (2013). J. Polym. Sci., Part A: Polym. Chem..

[cit24] Krajnc P., Stefanec D., Pulko I. (2005). Macromol. Rapid Commun..

[cit25] Barbetta A., Massimi M., Devirgiliis L. C., Dentini M. (2006). Biomacromolecules.

[cit26] Barbetta A., Cameron N. R. (2004). Macromolecules.

[cit27] Štefanec D., Krajnc P. (2007). Polym. Int..

[cit28] Sherrington D. C., Cameron N. R. (1997). Macromolecules.

[cit29] Cameron N. R. (2005). Polymer.

[cit30] Oh B. H. L., Bismarck A., Chanpark M. B. (2014). Biomacromolecules.

[cit31] Kovačič S., Matsko N. B., Jerabek K., Krajnc P., Slugovc C. (2013). J. Mater. Chem. A.

[cit32] Gao H., Peng Y., Pan J., Zeng J., Song C., Zhang Y., Yan Y., Shi W. (2014). RSC Adv..

[cit33] Zheng X., Zhang Y., Wang H., Du Q. (2014). Macromolecules.

[cit34] Hua Y., Zhang S. M., Chen J. D., Zhu Y. (2013). J. Mater. Chem. A.

[cit35] Zhang S. M., Chen J. D. (2009). Chem. Commun..

[cit36] Zhu Y., Zhang S., Hua Y., Zhang H., Chen J. (2014). Ind. Eng. Chem. Res..

[cit37] Hua Y., Chu Y. Q., Zhang S. M., Zhu Y., Chen J. D. (2013). Polymer.

[cit38] Zhu Y., Zhang R. R., Zhang S. M., Chu Y. Q., Chen J. D. (2016). Langmuir.

[cit39] Sun G., Li Z., Ngai T. (2010). Angew. Chem..

[cit40] Li Z., Wei X., Ngai T. (2011). Chem. Commun..

[cit41] Zhang N., Zhong S., Chen T., Zhou Y., Jiang W. (2017). RSC Adv..

[cit42] Yi W., Wu H., Wang H., Du Q. (2016). Langmuir.

[cit43] Ikem V. O., Menner A., Bismarck A. (2010). Langmuir.

[cit44] Hu Y., Huang J., Zhang Q., Yang Y., Ma S., Wang C. (2015). RSC Adv..

[cit45] Zheng Z., Zheng X., Wang H., Du Q. (2013). ACS Appl. Mater. Interfaces.

[cit46] Sergienko A. Y., Tai H., Moshe N., Silverstein M. S. (2010). J. Appl. Polym. Sci..

[cit47] Xu H., Zheng X., Huang Y., Wang H., Du Q. (2016). Langmuir.

[cit48] Zhang S. M., Chen J. D. (2007). Polymer.

[cit49] Zhang S., Chen J., Perchyonok V. T. (2009). Polymer.

[cit50] Wang S., Li J., Qi M., Gao X., Wang W. J. (2017). Langmuir.

[cit51] Butler R., Hopkinson I., Cooper A. I. (2003). J. Am. Chem. Soc..

[cit52] Mengual O., Meunier G., Cayre I., Puech K., Snabre P. (1999). Talanta.

[cit53] Mengual O., Meunier G., Cayre I., Puech K., Snabre P. (1999). Colloids Surf., A.

[cit54] Kimmins S. D., Cameron N. R. (2011). Adv. Funct. Mater..

[cit55] Gurevitch I., Silverstein M. S. (2010). J. Polym. Sci., Part A: Polym. Chem..

